# Predictors of Home Discharge Among Patients Hospitalized for Acute Respiratory Diseases

**DOI:** 10.7759/cureus.99235

**Published:** 2025-12-14

**Authors:** Takeshi Yamazaki, Michiko Tsuchiya, Yukio Nagasaka, Shota Kotani, Koji Iwai, Kunihiko Anami, Jun Horie

**Affiliations:** 1 Department of Physical Therapy, Faculty of Health Sciences, Kyoto Tachibana University, Kyoto, JPN; 2 Department of Respiratory Medicine, Rakuwakai Otowa Hospital, Kyoto, JPN; 3 Rakuwakai Kyoto Respiratory Center, Rakuwakai Otowa Hospital, Kyoto, JPN; 4 Department of Physical Therapy, Faculty of Rehabilitation Science, Kobe International University, Kobe, JPN; 5 Department of Physical Therapy, Faculty of Rehabilitation, Seijoh University, Tokai, JPN; 6 Department of Rehabilitation, Faculty of Health Sciences, Naragakuen University, Nara, JPN

**Keywords:** activity of daily living, acute respiratory disease, barthel index, functional independence measure, home discharge

## Abstract

Background/purpose: Acute respiratory diseases such as chronic obstructive pulmonary disease and pneumonia often result in functional decline and reduced likelihood of home discharge, yet early predictors of discharge outcomes remain unclear. This study aimed to examine the predictors of discharge of patients with acute respiratory diseases who have been admitted.

Methods: The data of 85 patients with acute respiratory diseases who had been admitted to the hospital were analyzed. The variables assessed included comorbid conditions, biochemical markers, frailty status, activities of daily living, and social background. The relationships between home discharge and these variables were evaluated using independent two-sample tests, multivariate logistic regression, and receiver operating characteristic curve analyses. Statistical significance was set at p < 0.05.

Results: The data from 49 participants who were discharged to their homes and nine participants who were not discharged to their homes were analyzed. Individuals who were discharged home were significantly younger (p = 0.044), had shorter hospital stays (p = 0.009), shorter rehabilitation durations (p = 0.020), and lower C-reactive protein levels (p = 0.020) than those who were not discharged home. They also had significantly higher admission Functional Independence Measure (FIM) (p = 0.002) and Barthel Index scores (p = 0.014). The admission FIM score was a significant predictor of home discharge (odds ratio = 1.05; 95% confidence interval = 1.01-1.09; p = 0.015). Receiver operating characteristic curve analysis revealed an area under the curve of 0.825 (p < 0.001) and an optimal cutoff score of 75.

Conclusion: Admission FIM scores may be associated with the probability of discharge of patients with acute respiratory diseases to their homes. These findings highlight the importance of early functional assessment in discharge planning and suggest that admission FIM scores may serve as a practical tool for optimizing patient-centered care pathways.

## Introduction

Respiratory conditions such as chronic obstructive pulmonary disease (COPD) and interstitial lung disease (ILD) are major contributors to morbidity and mortality globally and represent critical public health challenges [1]. Pneumonia and other lower respiratory tract infections disproportionately affect older adults and people with compromised immune systems [2], and they are closely associated with prolonged hospital stays and reduced rates of discharge to the home, especially for older adults [3,4]. Hospitalization increases the risk of functional decline, which often results in lower rates of discharge home and increased transfers to nursing homes or other healthcare facilities. Identifying factors associated with discharge destination is crucial for guiding rehabilitation interventions and allocating medical and welfare resources. Previous studies have identified decreased physical function, frailty, and comorbidities as factors associated with lower home discharge rates. Discharge planning is associated with postdischarge quality of life and the risk of hospital readmission, and consideration of the factors that enable home discharge may facilitate the development of effective rehabilitation strategies to prevent institutionalization and promote home-based support systems.

Several predictive models for discharge home have been developed for patients who have undergone surgery and individuals with stroke [5,6]. However, comparable models for patients with acute respiratory diseases are scarce. Some studies involving patients with pneumonia, including aspiration pneumonia, have reported physical disability at discharge, hypoalbuminemia at admission, tube feeding, and the need for oxygen therapy or suctioning at discharge as predictors of non-home discharge [7,8]. However, several of these factors are evaluated at or near the time of discharge, which limits their usefulness for early prediction of discharge outcomes during hospital stay. In clinical practice, patients are frequently admitted with diminished capacity for activities of daily living (ADLs), and this often hinders their discharge home.

Therefore, the purpose of this study was to examine the admission-related factors associated with home discharge of individuals hospitalized with acute respiratory diseases. We hypothesized that ADL function at admission is a stronger predictor of home discharge than indices of physical function, frailty, and comorbidities.

## Materials and methods

Ethical considerations

The study was performed in accordance with the principles outlined in the Declaration of Helsinki and was approved by the Ethics Committees of Kyoto Tachibana University and Rakuwakai Otowa Hospital (approval numbers: 24-65 and 23-00014). Participants were informed of the study objectives and procedures, and written informed consent was obtained.

Participants and covariates

This cross-sectional study was conducted from February 2022 to December 2024. The participants were 85 patients with acute respiratory diseases who were admitted to Rakuwakai Otowa Hospital from their homes.

Patients were included in this study if they were diagnosed with acute respiratory infections or acute exacerbations of COPD or ILD. On the other hand, they were excluded if they had severe comorbidities unrelated to respiratory illness, were admitted after transfer from another hospital with acute respiratory disease, or had advanced cognitive impairment.

**Figure 1:**
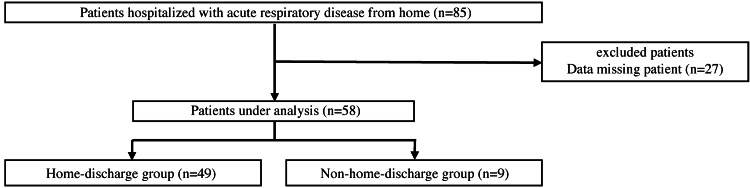
Flowchart of the study participants

The participants were allocated to the home-discharge (admitted from and discharged to home) and non-home-discharge (admitted from home but discharged to institutions or other hospitals) groups. Their assessment data were obtained on the day of admission or the following day, whenever feasible (Figure 1).

Frailty

Frailty status was determined using the Kihon Checklist, a 25-item yes/no questionnaire developed by the Ministry of Health, Labour and Welfare of Japan [9]. The checklist has seven domains: instrumental ADLs, physical capability, nutritional condition, oral health, social interaction/homebound tendency, cognitive ability, and depressive mood. Negative responses were scored, with higher scores indicating increased frailty risk. Participants who endorsed eight or more items were classified as frail.

The Kihon Checklist was used, which is an open-access, government-developed tool for frailty assessment. Previous research has demonstrated the reliability and validity of the Kihon Checklist for screening older adults at high risk of requiring long-term care. It has also been shown to predict disability and future long-term care dependence [10-12].

Comorbidities

The Charlson Comorbidity Index (CCI) was used to assess the comorbidities. It is widely accepted for classifying and quantifying prognostic comorbid conditions that may affect mortality and clinical outcomes [13]. It has demonstrated high reproducibility and predictive validity for outcomes such as mortality, hospital readmission, and healthcare resource utilization in various patient populations [14].

The CCI comprises 19 disease categories, each of which is assigned a weight based on the relative risk of mortality within one year. The total score is obtained by summing the weights of the individual conditions, with higher scores indicating a greater comorbidity burden. The individual weights are calculated based on the diagnostic information available at the time of admission. The CCI is freely available and does not require a license for use in research or clinical practice.

Assessment of ADLs

The ADL status of the participants was assessed using the Functional Independence Measure (FIM) and the Barthel Index (BI). These instruments are widely used in rehabilitation research, and their psychometric properties have been established. The FIM comprises 18 items covering motor and cognitive function. Each item is rated on a 7-point scale, and the total score ranges from 18 to 126. Higher scores indicate greater independence [15]. FIM scores were analyzed as numerical data without reproducing any specific items or scoring criteria; therefore, no license permission was required. On the other hand, the BI evaluates 10 fundamental physical activities, including feeding, grooming, bathing, dressing, toileting, and mobility. These activities are scored based on the assistance required, and the total score ranges from 0 to 100 points [16]. The BI is a freely available and widely used instrument that does not require license registration. Both measures have been validated in multiple clinical populations, including older adults and patients with neurological or musculoskeletal conditions [15,16].

The evaluations were conducted by licensed physical therapists at admission. The rationale for using both scales was to address a limitation of earlier research on aspiration pneumonia that used only the BI. The use of both instruments facilitated a comprehensive and detailed assessment of ADLs.

Blood biomarkers

Blood samples were obtained at admission to assess hematological and biochemical markers. The markers examined were total protein (TP), albumin (ALB), C-reactive protein (CRP), white blood cell count (WBC), and hemoglobin (Hb). They served as indicators of nutritional status, inflammatory activity, and anemia. TP is commonly used to assess nutritional status, with low concentrations indicating malnutrition or protein deficiency [17]. In contrast, CRP, WBC, and ALB concentrations are markers of inflammation; elevated CRP and WBC concentrations and decreased ALB concentrations indicate systemic inflammation or infection [18]. Hb is a standard biomarker for anemia, and low Hb concentrations are associated with reduced oxygen-carrying capacity and poor prognosis [19]. These blood biomarkers are clinically useful for evaluating the general condition of patients, informing treatment strategies, and predicting outcomes in acute care and rehabilitation settings.

Statistical analysis

The home-discharge status was the primary outcome. The sample size was determined a priori using G*Power 3.1.9.7 (Heinrich-Heine-Universität Düsseldorf, Düsseldorf, Germany). The parameters for the logistic regression analysis were an effect size (f²) of 0.15, a power of 0.80, and a significance level (α) of 0.05. The model included one main predictor and six covariates based on the hypothesis that the level of performance of ADLs at admission would have the greatest impact on discharge destination. The minimum required sample size was 55, and data from 58 participants were ultimately analyzed.

The admission characteristics associated with home discharge among patients admitted with acute respiratory diseases were identified through intergroup comparisons and logistic regression analyses. The normality of continuous and categorical variables was assessed using the Shapiro-Wilk test. For intergroup comparisons, independent two-sample t-tests were used for continuous variables, and χ² tests were used for categorical variables. Logistic regression was performed with the home-discharge status as the dependent variable and the admission FIM and BI scores, length of hospitalization, rehabilitation duration, and CRP concentrations as the independent variables. Age and sex were included as covariates. Variable selection was performed using a stepwise forward likelihood ratio approach [20]. The predictive value of the admission FIM score was further evaluated using receiver operating characteristic curve analysis, which provided the area under the curve (AUC) and cutoff score. Statistical significance was set at p < 0.05, and statistical analyses were performed using IBM Statistical Package for the Social Sciences Statistics, version 27 (IBM Corp., Armonk, NY).

## Results

Fifty-eight patients with acute respiratory diseases who were admitted to the hospital were included in the final study cohort. The median age was 81 years (interquartile range (IQR) = 73-85 years), the mean BMI was 19.5 kg/m^2^ (IQR = 17.2-21.8 kg/m^2^), and the median CCI score was 2 (IQR = 1-3). The other baseline characteristics are provided in Table 1.

**Table 1:** Characteristics of the study participants The data are shown as median (interquartile range) ^*^χ² test; all other analyses were performed using t-tests AECOPD: acute exacerbation of chronic obstructive pulmonary disease; AE-ILD: acute exacerbation of interstitial lung disease; BMI: body mass index; CCI: Charlson comorbidity index

Variables	All patients (n = 58)	Home-discharge group (n = 49)	Non-home-discharge group (n = 9)	p value	Effect size (φ,r)
Age (years)	81 (73-85)	77 (73-85)	82 (82-86)	0.044	0.264
Men, n (%)	38 (65.5)	31 (63.3)	7 (77.8)	0.476*	0.111
Height (m)	1.59 (1.5-1.65)	1.6 (1.52-1.65)	1.59 (1.5-1.59)	0.275	0.143
Body weight (kg)	47.9 (40.1-58.4)	47.7 (64-59.6)	49 (38.7-49.7)	0.264	0.147
BMI (kg/m^2^)	19.5 (17.2-21.8)	19.7 (17.3-22)	17.2 (15.1-20.6)	0.237	0.155
CCI (point）	2 (1-3)	2 (1-3)	3 (2-3)	0.137	0.195
Length of hospital stay (day）	17 (11-30)	16 (11-22)	25 (15-71)	0.009	0.342
Rehabilitation period (day)	14.5 (8.3-27)	13 (8-18)	21 (17-63)	0.02	0.305
Proportion of patients living alone, n (%)	11 (22.9)	20 (40.8)	1 (11.1)	0.135^*^	0.224
Proportion of patients certified for long-term care insurance, n (%)	24 (41.4)	18 (36.7)	6 (66.6)	0.142^*^	0.22
Number of hospitalizations within the past year (times)	0 (0-1)	0 (0-1)	0 (0-1)	0.783	0.036
Acute pneumonia, n (%)	29 (50)	24 (49)	5 (55.6)	0.915^*^	0.055
AECOPD, n (%)	16 (27.6)	14 (28.6)	2 (22.2)		
AE-ILD, n (%)	13 (22.4)	11 (22.4)	2 (22.2)		

The participants were stratified by discharge destination into the home-discharge (n = 49) and non-home-discharge (n = 9) groups. The group comparisons revealed that the patients discharged home were significantly younger (p = 0.044), had shorter durations of hospitalization (p = 0.009) and rehabilitation (p = 0.020), and lower CRP concentrations (p = 0.020) than those who were not. They also had significantly higher FIM (p = 0.002) and BI (p = 0.014) scores at admission (Tables 1, 2).

**Table 2:** Measurement results at admission The data are shown as median (interquartile range) Alb: albumin; BI: Barthel Index; CRP: C-reactive protein; FIM: functional independence measure; Hb: hemoglobin; TP: total protein; WBC: white blood cell count

Parameters	All patients (n = 58)	Home-discharge group (n = 49)	Non-home-discharge group (n = 9)	p value	Effect size (r)
Basic checklist (point)	12.5 (6.8-17)	1.59 (1.5-1.66)	1.59 (1.5-1.67)	0.22	0.16
FIM (point)	84 (53.5-103.3)	89 (53.5-104)	40 (30-70)	0.002	0.405
BI (point)	55 (23.8-80)	70 (35-80)	20 (0-35)	0.014	0.321
TP (g/dL)	6.7 (6.1-7.2)	6.7 (6.1-7.0)	6.8 (6.2-7.1)	0.774	0.038
Alb (g/dL)	3.3 (2.9-3.6)	3.3 (3.0-3.6)	3.0 (2.5-3.2)	0.06	0.247
CRP (mg/dL)	8.0 (1.4-14.1)	7.8 (1.3-13.6)	13.6 (12.7-26.9)	0.02	0.306
WBC (/μL)	9.4 (6.9-12.9)	8.8 (6.8-11.7)	11.6 (10.3-13.9)	0.076	0.233
Hb (g/dL)	12 (11.4-13.6)	12.1 (11.5-14.1)	11.6 (11.1-12.1)	0.179	0.176

**Figure 2:**
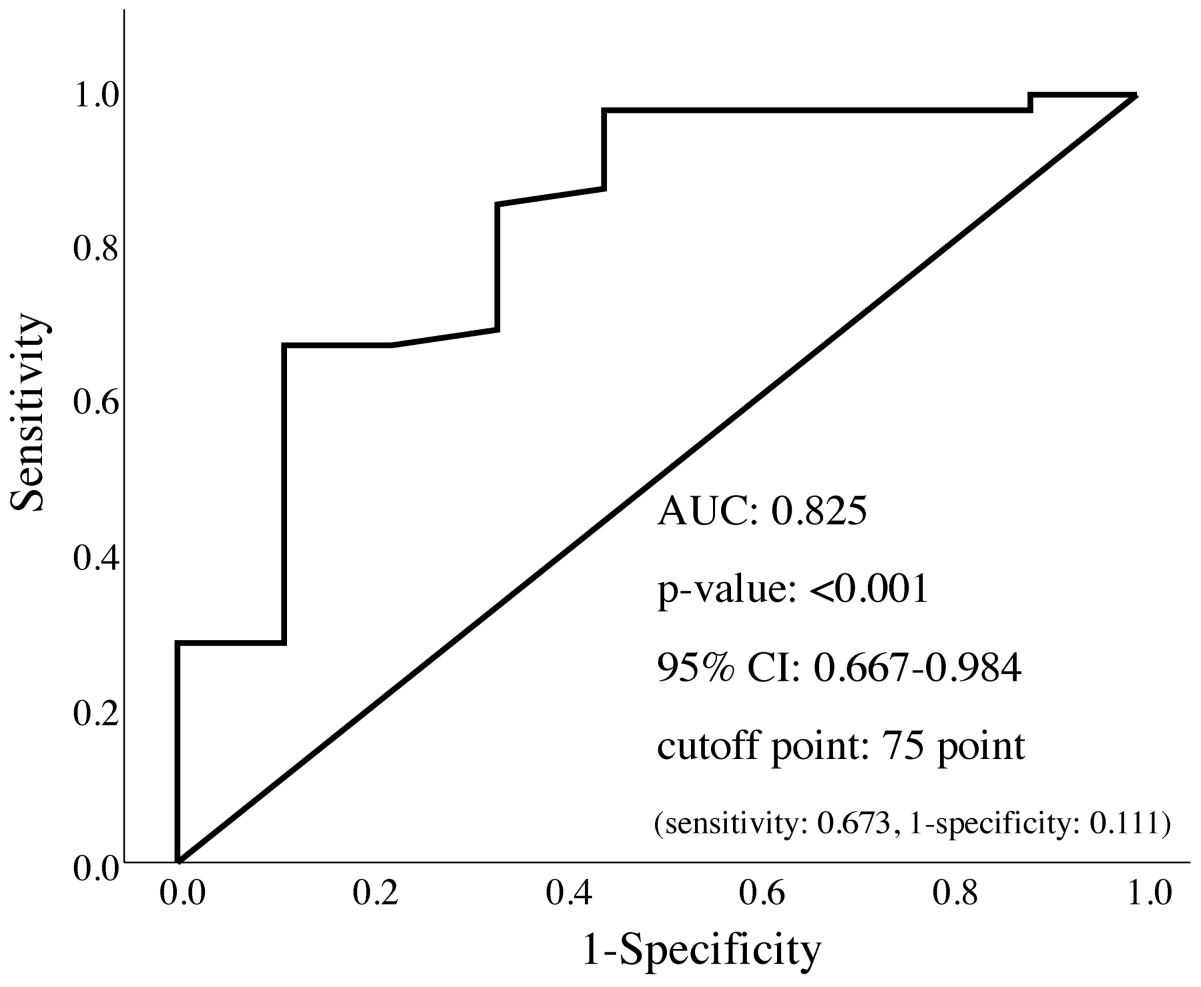
Receiver operating characteristic curve analysis results AUC: area under the curve; CI: confidence interval

Multivariable logistic regression identified the FIM score at admission as a significant predictor of home discharge (Table 3). Receiver operating characteristic curve analysis revealed that the admission FIM score predicted home discharge with an AUC of 0.825 (p < 0.001). The optimal cutoff value was 75 points (Figure 2).

**Table 3:** Logistic regression analysis of work availability Hosmer-Lemeshow: 0.176 Percentage of correct classifications: 87.5% 95% CI: 95% confidence interval for unstandardized coefficients; FIM: functional independence measure; OR: odds ratio

Measure	OR (95% CI)	p value
FIM	1.05 (1.01-1.09)	0.015

## Discussion

This study aimed to investigate the association between physical function at admission and the probability of discharge of patients admitted to the hospital for acute respiratory diseases to their homes. The patients were categorized into groups based on their discharge destination. Significant differences were observed among the groups in terms of age, length of hospital stay, rehabilitation period, CRP concentrations, and FIM and BI scores at admission. Multivariable logistic regression after controlling for age and sex and incorporating length of stay, rehabilitation days, FIM score, BI score, and CRP concentration as independent variables revealed that the FIM score at admission was independently associated with home discharge. Patients with an admission FIM score of 75 or higher had a higher likelihood of returning home. To the best of our knowledge, this is the first study to report an admission FIM cutoff score for predicting home discharge in this patient population.

Association between home discharge and FIM

The determination of discharge destination is influenced by several complex factors, including physical function and environmental and social support systems. Previous studies have identified age [7,21,22], disease severity [6,7,20,22], comorbidities [20], and frailty [23] as predictors of home discharge. However, these studies primarily focused on patients with orthopedic, cardiac, or cerebrovascular diseases, and evidence specific to patients with respiratory diseases remains limited.

The FIM, an assessment tool for ADLs, was a useful predictor of home discharge in the present study. It is a standardized scale that evaluates both motor and cognitive functions and provides a comprehensive reflection of the activity level and capacity for social participation of the patient [6]. The finding that higher FIM scores at admission were associated with a greater likelihood of home discharge highlights the utility of the FIM as an indicator of functional independence and self-management capabilities in the postdischarge living environment.

The FIM is widely used in acute care hospitals and convalescent rehabilitation settings, and its reliability as a standard clinical assessment tool has been well established. Therefore, predicting home discharge based on the FIM is a practical and reproducible approach. Only FIM was identified as a significant predictor of home discharge in this study, unlike the CCI and the presence of frailty. This highlights the central role of physical functional independence in determining discharge outcomes.

Patients with respiratory diseases often require continued care after discharge, including home oxygen therapy and outpatient follow-up. Accurate assessment of functional status using the FIM at admission may facilitate the planning of appropriate postdischarge support systems and the strengthening of community-based care coordination.

Direct association between FIM score and discharge destination

The literature consistently reports positive correlations between FIM scores and the likelihood of home discharge. Each incremental point increase in the FIM score has been associated with a 1.08-fold increase in the odds of home discharge for patients with stroke. Those with FIM scores ≥80 have a 12-fold higher likelihood of being discharged home [6]. ADL capacity has also been established as a significant predictor of institutional discharge of patients with hip fractures (odds ratio (OR) = 4.56; 95% confidence interval (CI) = 4.22-4.92) [21]. Surgical outcomes have also been influenced by functional status, especially postoperative ADL performance, such as self-care abilities [7].

Prehospitalization functional status has been reported as a strong determinant of discharge home in intensive care settings (OR = 7.10; 95% CI = 1.65-30.44) [4]. Our study also revealed significant associations between admission FIM scores and home discharge of patients with acute respiratory diseases. This underscores the clinical value of early ADL assessment during acute illness. The comorbidity burden and frailty status did not differ between the groups. However, the admission FIM score was more strongly associated with discharge destination than hospitalization or rehabilitation duration. These observations suggest that ADL assessment may be a more reliable predictor of home discharge. Future prospective trials should explore whether adjusting the rehabilitation intensity for patients with lower admission FIM scores can improve discharge outcomes.

Limitations

The cross-sectional design of this study precludes establishing causal relationships. The sample included patients with acute pneumonia, COPD exacerbations, and ILD exacerbations. In addition, a substantial imbalance in the number of participants between the two groups resulted in heterogeneity.

Discharge destination decisions are inherently multifactorial and complex and should not be based solely on functional outcome assessments. Biopsychosocial factors such as family structure, home environment, socioeconomic background, and insurance coverage substantially influence discharge planning. However, these factors are often difficult to capture comprehensively from existing clinical databases or electronic medical records, and they could not be fully captured in the present study.

Functional assessment tools such as the FIM and BI should be regarded as tools to support clinical decision-making during discharge planning rather than determinants of discharge destination. Discharge to home is not necessarily the ideal or safest option because some patients may require ongoing medical or caregiving support after returning home.

The FIM and other ADL assessments have been reported as useful indicators for objectively evaluating the functional status of a patient and predicting the degree of independence and care requirements after discharge for various disease populations. These tools enable healthcare professionals to implement evidence-based early discharge planning and provide optimal care and rehabilitation tailored to the needs of each patient.

## Conclusions

The objective of this study was to examine the predictors of home discharge among patients admitted to the hospital for acute respiratory diseases. The findings indicated that the home-discharge group had significantly lower age, shorter hospital stay and rehabilitation period, and lower CRP concentrations than the non-home-discharge group. They also had higher FIM and BI scores at admission. No group differences were observed related to comorbidity burden or frailty. Multivariate logistic regression analysis identified the admission FIM score as a significant factor. Patients with scores of ≥75 had a higher likelihood of home discharge. These findings highlight the importance of functional status at admission as a practical indicator for early discharge planning for patients with acute respiratory diseases and provide direction for clinical application and prospective research.
